# Initial treatment choices for long‐term remission of chronic insomnia disorder in adults: a systematic review and network meta‐analysis

**DOI:** 10.1111/pcn.13730

**Published:** 2024-08-26

**Authors:** Yuki Furukawa, Masatsugu Sakata, Toshiaki A. Furukawa, Orestis Efthimiou, Michael Perlis

**Affiliations:** ^1^ Department of Neuropsychiatry University of Tokyo Tokyo Japan; ^2^ Department of Neurodevelopmental Disorders Nagoya City University Graduate School of Medical Sciences Aichi Japan; ^3^ Department of Health Promotion and Human Behavior and of Clinical Epidemiology Kyoto University Graduate School of Medicine / School of Public Health Kyoto Japan; ^4^ Institute of Social and Preventive Medicine (ISPM) University of Bern Bern Switzerland; ^5^ Institute of Primary Health Care (BIHAM) University of Bern Bern Switzerland; ^6^ Behavioral Sleep Medicine Program, Department of Psychiatry University of Pennsylvania Philadelphia Pennsylvania USA; ^7^ Department of Psychiatry and The School of Nursing University of Pennsylvania Philadelphia Pennsylvania USA

**Keywords:** chronic insomnia disorder, cognitive behavioral therapy for insomnia, hypnotic, Insomnia

## Abstract

**Background:**

We aimed to evaluate the comparative efficacy and acceptability of cognitive behavioral therapy for insomnia (CBT‐I), pharmacotherapy, and their combination in the long and short terms among adults with chronic insomnia disorder.

**Methods:**

We searched multiple databases to December 27, 2023. We included trials in hypnotic‐free adults with chronic insomnia comparing at least two of CBT‐I, pharmacotherapy, or their combination. We assessed the confidence in evidence using CINeMA. The primary outcome was long‐term remission. Secondary outcomes included all‐cause dropout and self‐reported sleep continuity measures in the long term, and the same outcomes in the short term. We performed frequentist random‐effects network meta‐analyses (CRD42024505519).

**Findings:**

We identified 13 trials including 823 randomized participants (mean age, 47.8 years; 60% women). CBT‐I was more beneficial than pharmacotherapy in the long term (median duration, 24 weeks [range, 12 to 48 weeks]; remission odds ratio, 1.82 [95% confidence interval (CI), 1.15–2.87]; [certainty of evidence: high]), while there was weaker evidence of benefit of combination against pharmacotherapy (1.71 [95% CI, 0.88–3.30: moderate]) and no clear difference of CBT‐I against combination (1.07 [95% CI, 0.63–1.80: moderate]). CBT‐I was associated with fewer dropouts than pharmacotherapy. Short‐term outcomes favored CBT‐I over pharmacotherapy except total sleep time. Given the average long‐term remission rate in the pharmacotherapy‐initiating arms of 28%, CBT‐I resulted in a long‐term remission rate of 41% (95% CI, 31%–53%) and combination 40% (95% CI, 25%–56%).

**Interpretation:**

The current study found that starting with CBT‐I for chronic insomnia leads to better outcomes than pharmacotherapy. Combination may be better than pharmacotherapy alone, but unlikely to be worth the additional burden over CBT‐I alone.

Chronic insomnia is common and disabling.^1^ As many as 8% of the people in the United States used sleep medications in 2020,^2^ with this rate having doubled in the past decade.^3^ Around 20% of hypnotic users are prescribed sleeping aids for longer than 180 days.^4^, ^5^ This is concerning, given that a recent network meta‐analysis (NMA) found very sparse evidence supporting hypnotics in the long term.^6^ Another treatment option is cognitive behavioral therapy for insomnia (CBT‐I), a nonpharmacological intervention that is now recommended as the first‐line treatment^7^ and has been shown to be effective in the long term.^8^ Although many patients prefer nonpharmacological treatments over medications, nonpharmacological options are rarely provided.^9^ Factors impeding the dissemination of CBT‐I include not only the lack of clinicians' confidence in administering it but also the lack of knowledge among clinicians and patients regarding its comparative efficacy against pharmacological therapies.^10^

Another recent NMA suggested potential superiority of CBT‐I over pharmacotherapies (sleeping medications) and superiority of combination therapy (CBT‐I plus pharmacotherapies) over pharmacotherapies alone, for people with chronic insomnia with or without sleeping medications at the end of the acute‐phase treatment.^11^ However, this NMA included hypnotic‐resistant chronic insomnia and hence could not answer the clinical question of which treatment strategy to choose when starting to treat medication‐naive chronic insomnia. Moreover, it had important methodological limitations, such as violation of transitivity assumption (e.g. hypnotic users were included for pharmacotherapy versus combination comparison but excluded for comparisons including the psychotherapy‐alone arm), including nonpharmacological interventions not shown effective for chronic insomnia (e.g. sleep hygiene education and relaxation),^12^ and including medications not normally used for treating chronic insomnia (e.g. dexmedetomidine). Also, it could not provide conclusions about the long‐term comparative efficacy.

In the current study, we explored the long‐term relative efficacy and acceptability of CBT‐I, pharmacotherapy, and their combination as the initial treatment choice with the use of an NMA, focusing on trials that randomized people not currently on treatment for their chronic insomnia.

## Methods

We followed the Preferred Reporting Items for Systematic Reviews and Meta‐Analyses (PRISMA) guideline extension for NMA.^13^ This protocol was prospectively registered in PROSPERO (CRD42024505519) and can be found in eAppendix S2‐1.

### Data sources

#### Criteria for considering trials for this review

We included all randomized controlled trials that compared CBT‐I, pharmacotherapies, or their combination against each other in the treatment of hypnotic‐free adults with chronic insomnia. We included trials of patients of both sexes aged 18 years or older with chronic insomnia either diagnosed according to formal diagnostic criteria (such as the *Diagnostic and Statistical Manual of Mental Disorders*, the International Classification of Diseases, or the *International Classification of Sleep Disorders*) or judged so by clinical experts (e.g. presence of significant symptoms). The criteria needed to include significant distress or daytime impairment. We tested the effect of including studies without a formal diagnosis of chronic insomnia in a sensitivity analysis. We included patients with psychiatric or physical comorbidities. We excluded trials if patients currently using prescription or over‐the‐counter sleep medications were included. We excluded trials focusing on chronic insomnia not responsive to psychotherapy or pharmacotherapy for chronic insomnia, but included trials if patients discontinued the medications for a certain period before randomization. We regarded CBT‐I as a psychotherapy involving any one of the following components shown effective in a recent component NMA^12^: sleep restriction, stimulus control, cognitive restructuring, and third‐wave components (mindfulness and acceptance and commitment therapy) (eAppendix S2‐1). We included drugs that were proven to be effective in a recent NMA^6^ (benzodiazepines, doxylamine, eszopiclone, lemborexant, seltorexant, suvorexant, trazodone, zaleplon, zolpidem, zopiclone). When treatments were provided in sequence (e.g. pharmacotherapy first, then CBT‐I), we categorized the intervention according to the initial treatment strategy (e.g. pharmacotherapy, in this case). Where multiple arms were reported in a single trial, we included only the relevant arms.

### Search methods for identification of studies

We performed a comprehensive literature search in PubMed, CENTRAL, and PsycINFO from database inception to 27 December 2023. We used a combination of index and free terms of psychological and pharmacological treatments and insomnia with filters for randomized clinical trials (eAppendix S2‐1). We also searched the World Health Organization's International Clinical Trials Registry Platform. We imposed no date, language, or publication status restriction at the search stage, but we included only trials in English at the screening stage. We checked the reference lists of identified studies and review articles for additional potentially eligible records.

### Data collection and analysis

#### Selection of studies

Two review authors (Y.F. and M.S.) independently screened titles and abstracts of all potential studies we identified in our systematic search. We retrieved the full‐text study reports, and two review authors independently screened the studies for inclusion and recorded reasons for exclusion of the ineligible studies. We resolved any disagreement through discussion. We identified publications from the same study so that each study rather than each report is the unit of analysis in the review. We assessed the interrater reliability of the full‐text screening decisions with Cohen κ and percentage agreement.

### Data items

Two review authors (Y.F. and M.S.) independently extracted data from the included studies. Any disagreement was resolved through discussion. We assessed the included trials using the revised risk‐of‐bias tool by Cochrane.^14^ Any disagreement was resolved through discussion. We measured the interrater reliability of the overall risk‐of‐bias assessment with Cohen κ and percentage agreement, and that of the extracted primary outcomes with intraclass correlation.

### Primary outcome and secondary outcomes

The primary outcome of interest was treatment remission, defined as reaching a satisfactory state, measured by any validated self‐reported scale (e.g. Insomnia Severity Index [ISI] ≤7, the Pittsburgh Sleep Quality Index ≤5, sleep efficiency ≥85%, sleep latency ≤30 min) at long‐term follow‐up (longest follow‐up between 3 and 12 months). We prioritized intention‐to‐treat analyses whenever possible. When original publications did not report the number of remitters, we imputed remission based on continuous outcomes using a previously validated method.^12^, ^15^ Secondary outcomes included all‐cause dropouts (as a proxy measure of acceptability), various self‐reported sleep continuity measures, including sleep efficiency (%), total sleep time (minutes), sleep latency (minutes) and wake after sleep onset (minutes). We also examined short‐term outcomes (outcomes at posttreatment of the first‐step treatment phase). We used odds ratios (ORs) for analyzing dichotomous outcomes.^12^, ^16^ We translated the ORs into the experimental event rates using the weighted mean proportion of remitters in the pharmacotherapy‐initiating arms as the control event rate, aiming to improve interpretability.^17^, ^18^ We used the mean difference (MD) for continuous outcomes expressed in minutes and percentages, and standardized MDs (SMDs) for continuous outcomes measured in variable scales.

### Statistical analysis

We created a network diagram to visualize the available evidence. Transitivity is a fundamental assumption behind NMA.^19^ Transitivity implies that we can combine the direct evidence from A versus C and B versus C studies to learn indirectly about the comparison A versus B. This, however, will be questionable if there are important differences in the distribution of the effect modifiers across treatment comparisons. To assess transitivity, we created box plots of trial and patient characteristics deemed to be possible effect modifiers (publication year, age, and baseline severity) and visually examined whether they were similarly distributed across treatment comparisons. We checked consistency using global (design‐by‐treatment) and local (back‐calculation) tests.^20^, ^21^ Given the expected variability in the patients and treatments to be included, we conducted a random‐effects NMA. We visualized NMA results using pharmacotherapy as reference and ordering treatments according to P‐scores, which provide an overall ranking of treatments.^22^

We assessed heterogeneity by looking at the SD of random effects (τ^2^) and comparing it against empirical distributions,^23^ and by creating prediction intervals.^24^ We assessed possible reporting bias and small‐study effects using contour‐enhanced funnel plots when ≥10 trials were available for a single comparison. We assessed certainty of evidence using CINeMA.^25^

We performed prespecified sensitivity analyses on the primary outcome to examine the influence of including studies with informal diagnostic criteria, comorbidities, high dropout rates, and high overall risk of bias. In addition, we conducted a post hoc sensitivity analysis excluding arms in which new treatment strategies were provided in the second step (e.g. pharmacotherapy first, then CBT‐I or combination) to examine the potential impact of carryover effect, and another with a stringent definition (e.g. acute CBT‐I followed by postacute combination therapy, acute pharmacotherapy followed by CBT‐I) to see whether any certain sequence outperformed others.

We conducted analyses in *R* ^26^ using *netmeta*,^27^ and *meta* ^28^ packages.

## Results

We identified 560 records for the title and abstract screening, then assessed 111 full texts. We included nine trials and 627 participants for the primary outcome (long‐term follow‐up) and 13 trials with a total of 823 participants for the posttreatment assessment (eAppendix S2‐2). The interrater reliability of judgments for full‐text screening was substantial, with a κ of 0.65 (95% confidence interval [CI], 0.51 to 0.79) and percentage agreement of 84%. The eAppendix S2‐2 lists the included and excluded trials.

Typical participants were middle‐aged with moderate insomnia symptoms (mean age, 47.8 years [SD, 13.5 years], based on 12 trials; 510 of 823 [62%] were female, reported in 13 trials; and the baseline ISI score was 17.4 [SD, 4.0], reported in six trials). Twelve trials used formal operationalized criteria. Nine trials had two arms and four trials had three arms according to our categorization (11 CBT‐I–initiating arms, eight combination‐initiating arms, 11 pharmacotherapy‐initiating arms). Of the 19 arms including CBT‐I components, 18 arms included stimulus control, 17 sleep restriction, 15 cognitive restructuring, and one third‐wave components. Of 19 arms including pharmacotherapy, six arms used zolpidem (5–10 mg), four temazepam (7.5–30 mg), four zopiclone (3.75–7.5 mg), two trazodone (50–150 mg), two triazolam (0.25 mg), and one eszopiclone (3 mg). Nine trials reported outcomes at long‐term follow‐up (median duration, 24 weeks [range, 12 to 48 weeks]) and 13 trials at posttreatment (median, 8 weeks [range, 2 to 12 weeks]). Two trials^29^, ^30^ used a sequential design and the rest used a parallel design. Tables 1, 2, 3 show the characteristics of the included trials.

**Table 1:** Characteristics of the included trials

	Value	Trials
Age, mean (SD), year	47.8 (13.5)	12
Sex, No. (%)		
Female	510/823 (62%)	12
Male	313/823 (38%)	12
Baseline severity (ISI score), mean (SD)	17.4 (4.0)	6
Diagnosis		
Formal operationalized criteria		12
Others		1
Region		
North America		8
Europe		3
Asia		2
Publication year, mean (range)	2009 (1993–2020)	13
No. of arms		
Total	30 Arms	13
CBT‐I–initiating	11 Arms	11
Combination‐initiating	8 Arms	8
Pharmacotherapy‐initiating	11 Arms	11
Delivery method		
Individual		8
Group		5
Self‐help		0

Abbreviations: CBT‐I, cognitive behavioral therapy for insomnia; ISI, Insomnia Severity Index; SD, standard deviation.

**Table 2:** Study and patient characteristics

Study	Country	Recruitment	Study center	Diagnosis	Intervention	Number	Age (year)	Female (*n*)
Included in the primary analysis								
Gross *et al*., 2011^40^	United States	Community	Single	Formal	CBT‐I	20	47.0	15
					Pharmacotherapy	10	53.5	7
Jacobs *et al*., 2004^41^	United States	Community	Single	Formal	CBT‐I	15	47.1	10
					Combination	18	49.1	12
					Pharmacotherapy	15	45.4	11
Morin *et al*., 1999^35^	Canada	Community	Single	Formal	CBT‐I	18	64.4	13
					Combination	20	65.2	13
					Pharmacotherapy	20	64.1	9
Morin *et al*., 2009^29^	Canada	Community	Single	Formal	CBT‐I	80	51.7	50
					Combination	80	48.8	47
Morin *et al*., 2020^30^	Canada, United States	Community	Multiple	Formal	CBT‐I	104	45.9	64
					Pharmacotherapy	107	45.4	68
Siversten *et al*., 2006^42^	Norway	Community	Single	Formal	CBT‐I	18	59.8	7
					Pharmacotherapy	18	61.3	6
Vallieres *et al*., 2005^43^	Canada	Community	Single	Formal	CBT‐I	6	41.6	3.3
					Combination	5	41.6	3.3
					Pharmacotherapy	6	41.6	3.3
Vgotzas *et al*., 2020^44^	United States	Community	Single	Others	CBT‐I	12	45.9	7
					Pharmacotherapy	12	44.6	6
Wu *et al*., 2006^45^	China	Community	Single	Formal	CBT‐I	19	38.0	10
					Combination	19	38.0	10
					Pharmacotherapy	20	38.0	11
Included only in the posttreatment analysis								
Mao *et al*., 2018^46^	China	Outpatients	Single	Formal	Combination	52	43.3	38
					Pharmacotherapy	52	42.5	36
Milby *et al*., 1993^37^	United States	Community	Single	Formal	Combination	8	32.5	4
					Pharmacotherapy	7	32.5	4
Pchelina *et al*., 2017^47^	Russia	Outpatients	Single	Formal	CBT‐I	23	47.5	16
					Pharmacotherapy	19	55.0	11
Zavesicka *et al*., 2008^48^	Czech	Outpatients	Single	Formal	CBT‐I	10	48.6	8
					Combination	10	46.1	7

Abbreviation: CBT‐I, cognitive behavioral therapy for insomnia.

**Table 3:** Intervention characteristics

Study	Number	Initial treatment	Weeks	Second‐step treatment	Follow‐up (weeks)	Sequence
Included in the primary analysis						
Gross *et al*., 2011^40^	20	tw	8	Naturalistic	20	CBT‐I → nat
	10	Eszopiclone 3 mg		As‐needed (12 w)		Pha → nat
Jacobs *et al*., 2004^41^	15	sr, sc, cr	8	Naturalistic	48	CBT‐I → nat
	18	sr, sc, cr, zolpidem 10 mg*		Naturalistic		Com → nat
	15	Zolpidem 10 mg*		NA	NA	NA
Morin *et al*., 1999^35^	18	sr, sc, cr	8	Naturalistic	48	CBT‐I → nat
	20	sr, sc, cr, temazepam 7.5–30 mg		Naturalistic		Com → nat
	20	Temazepam 7.5–30 mg		Naturalistic		Pha → nat
Morin *et al*., 2009^29^	80	sr, sc, cr	6	CBT‐I† or NT	24	CBT‐I → CBT‐I, CBT‐I → nat
	80	sr, sc, cr, zolpidem 10 mg		CBT‐I† or CBT‐I† + zolpidem 10 mg as‐needed		Com → CBT‐I, Com → Com
Morin *et al*., 2020^30^	104	sr, sc	6	Zolpidem or cr for 6 wk, then naturalistic	48	CBT‐I → Pha, CBT‐I → CBT‐I
	107	Zolpidem 5–10 mg		sr + sc or trazodone 50–150 mg for 6 wk, then naturalistic		Pha → CBT‐I, Pha → Pha
Siversten *et al*., 2006^42^	18	sr, sc, cr	6	Naturalistic	24	CBT‐I → nat
	18	Zopiclone 7.5 mg		Zopiclone 7.5 mg as‐needed		Pha → nat
Vallieres *et al*., 2005^43^	6	sr, sc, cr	10	sr, sc, cr, then naturalistic	12	CBT‐I → CBT‐I
	5	sr, sc, cr + zopiclone 3.75–7.5 mg		sr, sc, cr, then naturalistic		Com → CBT‐I
	6	Zopiclone 3.75–7.5 mg		sr, sc, cr + zopiclone 3.75–7.5 mg, then naturalistic		Pha → Com
Vgotzas *et al*., 2020^44^	12	sr, sc, cr	12	Naturalistic	24	CBT‐I → nat
	12	Trazodone 50–100 mg		Trazodone 50–100 mg		Pha → Pha
Wu *et al*., 2006^45^	19	sr, sc cr	8	Naturalistic	32	CBT‐I → nat
	19	sr, sc cr, temazepam 7.5–30 mg		Naturalistic		Com → nat
	20	Temazepam 7.5–30 mg		Naturalistic		Pha → nat
Included only in the post‐treatment analysis						
Mao *et al*., 2018^46^	52	sr, sc, cr, zolpidem 5–10 mg	8	NA	NA	NA
	52	Zolpidem 5–10 mg		NA		
Milby *et al*., 1993^37^	8	sc, triazolam 0.25 mg*	5	NA	NA	NA
	7	Triazolam 0.25 mg*		NA		
Pchelina *et al*., 2017^47^	23	sr, sc	2	NA	NA	NA
	19	Zopiclone 7.5 mg		NA		
Zavesicka *et al*., 2008^48^	10	sr, sc, cr	8	NA	NA	NA
	10	sr, sc, cr, trazodone 100 mg		NA		

Abbreviations: CBT‐I †, extended CBT‐I (monthly for 6 months); CBT‐I, cognitive behavioral therapy for insomnia; Com, combination; cr, cognitive restructuring; NA, not applicable; nat, naturalistic; NT, no treatment; Pha, pharmacotherapy; sc, stimulus control; sr, sleep restriction. *Gradual tapering.

Interrater reliability of extracted primary outcomes was almost perfect, with an intraclass correlation of 0.95 (95% CI, 0.92–0.97). The overall risk of bias for the primary outcome according to the Cochrane revised risk‐of‐bias tool was low in two of nine trials (22%), some concerns in four (44%), and high in three (33%) (eAppendix S2‐2). The interrater reliability for the overall risk of bias was moderate, with a squared weighted κ of 0.41 (95% CI, 0.03 to 0.78) and percentage agreement of 33%.

**Figure 1:**
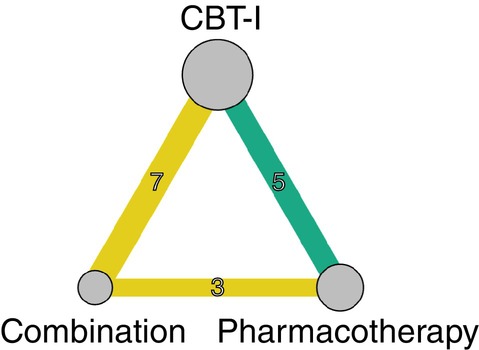
Network diagram for the primary outcome. The size of the nodes corresponds to the number of participants randomized to the treatment. The width of lines connecting treatments corresponds to the number of trials. This number is also shown on each line. Colors indicate the confidence in the evidence: green = high, yellow = moderate. CBT‐I = cognitive behavioral therapy for insomnia.

**Figure 2:**
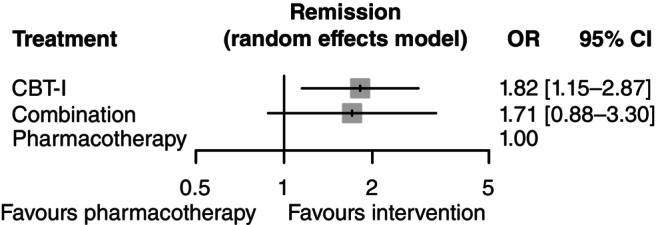
Results of network meta‐analysis for remission in the long term. CBT‐I, cognitive behavioral therapy for insomnia; CI, confidence interval; OR, odds ratio.

Figure 1 shows the network for the primary outcome and Fig. 2 the result of the NMA. eAppendix S2‐3 shows the assessment of transitivity, which found that potential effect modifiers were evenly distributed across comparisons. The global (design‐by‐treatment) test showed some evidence of inconsistency (*P* = 0.06), but the local (back‐calculation) method did not find disagreements between direct and indirect comparisons (eAppendix S2‐3). Heterogeneity of the primary outcome was limited (τ^2^ = 0.02), which was smaller than the majority of the existing meta‐analyses of mental health indicators comparing nonpharmacological interventions against pharmacological interventions.^23^ There was weak evidence of discrepancies between direct and indirect comparisons for CBT‐I versus combination and CBT‐I versus pharmacotherapy comparisons, but the indirect estimates were imprecise and the prediction intervals incorporating inconsistency did not meaningfully change the overall interpretation of results (eAppendix S2‐3). We did not evaluate publication bias and small‐study effects using funnel plots due to the limited number of trials. Figure 1 shows the result of NMA for the primary outcome and eAppendix S2‐4 shows the results of the pairwise meta‐analyses and the league tables. eAppendix S2‐6 shows the result of CINeMA for the primary outcome.

**Figure 3:**
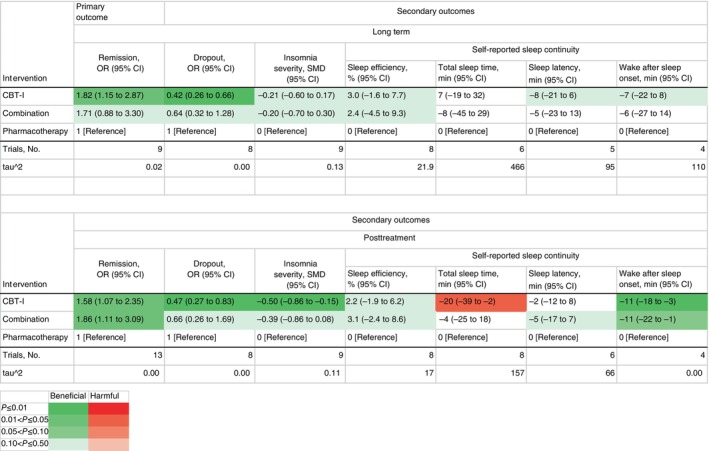
Results of network meta‐analyses for primary and secondary outcomes. CBT‐I, cognitive behavioral therapy for insomnia; CI, confidence interval; SMD, standardized mean difference.

Figure 3 tabulates the results of network meta‐analyses for the primary and secondary outcomes. We applied the Kilim plot,^31^ coloring cells in shades of green and red, according to the strength of statistical evidence against the null. We found evidence that initiating the treatment with CBT‐I (nine arms, *n* = 292) was more effective than with pharmacotherapy (seven arms, *n* = 193) in the long term (OR, 1.82 [95% CI, 1.15 to 2.87; certainty of evidence: high]). We also found weaker evidence of superiority of combination (five arms, *n* = 142) over pharmacotherapy alone (OR, 1.71 [95% CI, 0.88 to 3.30: moderate]). We did not find evidence of superiority of CBT‐I over combination (OR, 1.07 [95% CI, 0.63 to 1.80: moderate]).

CBT‐I was more beneficial than pharmacotherapy in various secondary outcomes; dropout in the long term (OR, 0.42 [95% CI, 0.26 to 0.66]), remission at posttreatment (OR, 1.58 [95% CI, 1.07 to 2.35]), dropout at posttreatment (OR, 0.47 [95% CI, 0.27 to 0.83]), insomnia severity at posttreatment (SMD, −0.50 [95% CI, −0.86 to −0.15]), and wake after sleep onset at posttreatment (MD, −11 min [95% CI, −18 to −3]). However, CBT‐I led to shorter total sleep time at posttreatment than pharmacotherapy (MD, −20 min [95% CI, −39 to −2]). The combination was better than pharmacotherapy alone in the remission at posttreatment (OR, 1.86 [95% CI, 1.11 to 3.09]) and wake after sleep onset at posttreatment (MD, −11 min [95% CI, −22 to −1]). There was no clear evidence of a difference between CBT‐I and combination.

Sensitivity analyses generally confirmed the superiority of CBT‐I over pharmacotherapy. The post hoc sensitivity analysis excluding arms in which new treatment strategies were provided in the second step were in line with the primary analysis. The post hoc sensitivity analysis with more stringent categorization suggested that the treatment strategies starting with CBT‐I and the treatment strategy starting with combination and then CBT‐I alone were more beneficial than the treatment strategy starting with pharmacotherapy alone with naturalistic follow‐up (eAppendix S2).

Given the weighted average proportion of remitters in pharmacotherapy‐initiating arms in the long term at 28%, we estimated that CBT‐I led to remission in 41% (95% CI, 31% to 53%) and combination in 40% (95% CI, 25% to 56%) of the patients. The weighted average proportion of dropouts in pharmacotherapy‐initiating arms in the long term was estimated to be 39%. Using this number, we estimated that CBT‐I led to dropouts in 21% (95% CI, 14% to 30%) and combination in 29% (95% CI, 17% to 45%) of the patients (Table 4).

**Table 4:** Estimated event rates for each condition

	Long term		Posttreatment	
	Remission, % (95% CI)	Dropouts, % (95% CI)	Remission, % (95% CI)	Dropouts, % (95% CI)
CBT‐I	41 (31 to 53)	21 (14 to 30)	38 (29 to 48)	8 (5 to 14)
Combination	40 (25 to 56)	29 (17 to 45)	42 (30 to 55)	11 (5 to 24)
Pharmacotherapy	28 [Reference]	39 [Reference]	28 [Reference]	16 [Reference]

Abbreviations: CBT‐I, cognitive behavioral therapy for insomnia; CI, confidence interval.

## Discussion

To our knowledge, we performed the first systematic review and NMA of the initial treatment choices for chronic insomnia, aiming to identify which treatment may maximize the chance of remission in the long term. Our findings showed that starting with CBT‐I was superior to starting with pharmacotherapy both in the long term and at the end of the acute treatment phase, both in terms of efficacy and acceptability. Combination therapy may be more effective and acceptable than pharmacotherapy alone in the short term, but there was no evidence of its superiority over CBT‐I alone. Total sleep time at posttreatment was shortest in CBT‐I at posttreatment, but the difference was unclear in the long term.

Based on these findings, we suggest people start chronic insomnia treatment with CBT‐I alone. Combining sleep medication with CBT‐I may be as effective as CBT‐I alone, but it entails more cost and possible side effects, such as residual sedation,^6^ dependence/withdrawal,^32^ and falls.^33^ In the case of pregnant women, there is also an elevated risk for miscarriage.^34^ However, given the short total sleep time in the CBT‐I arms at posttreatment, patients who are vulnerable to sleep loss may prefer starting with combination or pharmacotherapy. Some may find CBT‐I burdensome and prefer pharmacotherapy alone.

We confirmed the superiority of CBT‐I over pharmacotherapy alone for patients with hypnotic‐free chronic insomnia at posttreatment as previously suggested in people with chronic insomnia in general.^11^ The previous NMA did not support the long‐term superiority of any treatment over another because of the limited numbers of trials included.^11^ This may be because they categorized the long‐term follow‐ups in three categories (1–3, 6–8, and 12–24 months) and lost the statistical power to detect a difference even though they all tended to favor CBT‐I. We reasoned that comparative effectiveness was likely to remain stable in the long term,^35^ and therefore prespecified in the protocol to use the longest follow‐up in 3 to 12 months for the long‐term follow‐up outcome. Another strength of our study is that we defined CBT‐I as those including effective components^12^ and pharmacotherapies as those shown effective^6^ so that comparing them would be clinically relevant.

The clinical practice guideline of the American College of Physicians recommends CBT‐I as the initial treatment for chronic insomnia based on a series of pairwise meta‐analyses of active versus control conditions that investigated the efficacy and safety profile of treatments.^7^ Our findings further strengthen this recommendation by providing evidence on comparative efficacy and acceptability based on network meta‐analyses. Given patients' preference of nonpharmacological therapy over pharmacotherapy,^9^ clinicians, policymakers, and reimbursement bodies should take actions to make CBT‐I more widely accessible, so that patients' preferences can be respected in everyday practices.

Our study has several limitations. First, our study has the potential for sampling bias inherent in randomized controlled trials, specifically the selection bias of participants. Individuals who participate in intervention studies are often highly motivated,^36^ which could have a significant impact, particularly on psychological interventions. Furthermore, only one trial^37^ established a psychological placebo (e.g. providing sleep‐related information). In studies directly comparing psychological interventions and pharmacotherapy, expectations for psychotherapy^38^ and aversion to pharmacotherapy^9^ may have also influenced the results. Although there is no clear evidence of treatment preference of psychotherapy against pharmacotherapy being an effect modifier,^39^ caution should be exercised when interpreting the results, especially when patients prefer pharmacotherapy. Second, one may question the approach of combining all hypnotics shown effective by De Crescenzo *et al*.^6^ as “pharmacotherapy,” as they have different modes of action and their effectiveness for insomnia may also differ. We took this approach because the effect sizes shown in the NMA by De Crescenzo *et al*.^6^ appeared relatively similar among compounds. Moreover, the majority of hypnotics used in the included trials were benzodiazepines or Z‐drugs, which share a common mechanism of action. Still, it should be stressed that none of the trials used dual orexin receptor antagonists, and whether the findings apply to these new hypnotics is yet to be evaluated. However, given the relatively mild efficacy of dual orexin receptor antagonists in the short term and its sparse long‐term evidence,^6^ CBT‐I should remain the best initial treatment option until proven otherwise. Third, all of the CBT‐I programs were provided by therapists. Self‐help CBT‐I, such as internet CBT‐I, may be one solution to scale up its availability, but it remains unclear whether its effectiveness is comparable to therapist‐guided CBT‐I. Fourth, although the study found that CBT‐I and combination therapy are more beneficial than pharmacotherapy both in the long term (median, 24 weeks of follow‐up) and at posttreatment (median, 8 weeks), the relative effectiveness in the shorter period (several days after initiating the treatment) remains unclear. Fifth, most trials were conducted in North America and Europe, and the generalizability of the findings to other regions including Asia should be tested further in future studies.

## Conclusion

We found evidence that initiating treatment for chronic insomnia in adults with CBT‐I leads to more beneficial results compared with starting with pharmacotherapy alone. While combining CBT‐I and pharmacotherapy might be more effective than pharmacotherapy alone, we did not find evidence that would justify the use of combination therapy over using CBT‐I alone. Healthcare providers, policymakers, and insurers should make CBT‐I more widely accessible, so that patients' preferences can be respected in everyday practices.

## Disclosure statement

Y.F. has received consultancy fee from Panasonic and lecture fee from Otsuka outside the submitted work. M.S. is employed by the donation from the City of Nagoya. M.S. reports personal fees from SONY outside submitted work. T.A.F. reports personal fees from Boehringer‐Ingelheim, Daiichi Sankyo, DT Axis, Kyoto University Original, Micron, Shionogi, SONY, and UpToDate, and a grant from DT Axis and Shionogi, outside the submitted work. In addition, TAF has a patent 7,448,125 and a pending patent 2022–082495, and has licensed intellectual properties for Kokoro‐app to Mitsubishi‐Tanabe. T.A.F. is a statistical advisor of *Psychiatry and Clinical Neurosciences* and a co‐author of this article. To minimize bias, he was excluded from all editorial decision‐making related to the acceptance of this article for publication. M.P. wrote treatment manuals and books for CBT‐I, teaches CBT‐I, and is a founder of Hypknowledge LLC. O.E. has nothing to declare.

## Author contributions

Y.F. contributed to the conceptualization, methodology, project administration, formal analysis, data curation, interpretation, writing‐original draft, writing–review, and editing and visualization. M.S. contributed to the conceptualization, methodology, data curation, and writing–review and editing. T.A.F. contributed to the conceptualization, methodology, formal analysis, interpretation, writing–review and editing, and visualization. O.E. contributed to the methodology, formal analysis, interpretation, writing–review and editing, and visualization. M.P. contributed to the conceptualization, interpretation, writing–review and editing, and supervision. Y.F. and M.S. had full access to all of the data in the study and takes responsibility for the integrity of the data and the accuracy of the data analysis.

## Supporting information


**Data S1.** Prisma.

**Data S2.** Supporting Information.

## Data Availability

Y.F. had full access to all of the data in the study and takes responsibility for the integrity of the data and the accuracy of the data analysis. Codes for all analyses are available in a repository on GitHub (https://github.com/ykfrkw/W2I).
